# Educational quality may be a closer correlate of cardiometabolic health than educational attainment

**DOI:** 10.1038/s41598-022-22666-3

**Published:** 2022-10-27

**Authors:** Jenny M. Cundiff, Shayne S.-H. Lin, Robert D. Faulk, Ian M. McDonough

**Affiliations:** University of Alabama, Tuscaloosa, AL USA

**Keywords:** Psychology, Risk factors

## Abstract

Educational quality may be a closer correlate of physical health than more commonly used measures of educational attainment (e.g., years in school). We examined whether a widely-used performance-based measure of educational quality is more closely associated with cardiometabolic health than educational attainment (highest level of education completed), and whether perceived control (smaller sample only), executive functioning (both samples), and health literacy (smaller sample only) link educational quality to cardiometabolic health. In two samples (N = 98 and N = 586) collected from different regions of the US, educational quality was associated with cardiometabolic health above and beyond educational attainment, other demographic factors (age, ethnoracial category, sex), and fluid intelligence. Counter to expectations, neither perceived control, executive function, nor health literacy significantly mediated the association between educational quality and cardiometabolic health. Findings add to the growing literature suggesting that current operationalizations of the construct of education likely underestimate the association between education and multiple forms of health. To the extent that educational programs may have been overlooked based on the apparent size of associations with outcomes, such actions may have been premature.

## Testing the unique effect of educational quality on BMI and central adiposity in adulthood and hypothesized mediators

Major disparities in health by socioeconomic status persist in the Unites States despite enormous health care expenditures and decades of codified commitment to reducing health disparities^1^. Education is one indicator of socioeconomic status that has been consistently associated with multiple indicators of health over the life course^2,3^, including cardiometabolic disease and risk factors for poor cardiometabolic health such as greater BMI and central adiposity^4,5^. However, common measures of education used to inform policy and interventions on health outcomes may vastly underestimate the importance of education. The present study examines individual-level differences in educational quality and physical health outcomes beyond traditional measures of education in two diverse community samples. Additionally, we tested multiple mechanisms hypothesized to explain why increased education may result in better physical health.

### Does educational quality matter?

Although empirical evidence has largely examined educational *attainment* (e.g., years of education or highest degree attained) in relation to health, educational *quality* is likely a better measure of the construct of education in health research. Preliminary evidence suggests incremental predictive utility of educational quality above and beyond educational attainment^6,7^. Educational quality might be a stronger predictor of health compared to assessments of educational attainment because it more directly measures cognitive and skill-based benefits of both formal and informal education that are thought to confer benefits to health^8^. For example, education is hypothesized to influence cardiovascular risk, in part, through the acquisition of skills and abilities that lead to better income and employment, increased health literacy and general knowledge, and greater self-efficacy and perceived control^9–11^. Not all individuals that attain a higher level of education also experience the same gains in such skills and abilities. Further, educational quality may be a more useful measure for understanding educational disparities in health across race and socioeconomic status in the United States, given that similar quantities of educational attainment (e.g., years in school) tend to result in lower educational quality for racial and ethnic minority students^12^. As Sentell, Zhang and Ching stated, “Many minority adults thus get less ‘education’ for their years of schooling, ” suggesting that measures of educational attainment do not fully capture racial and ethnic disparities in schooling that may influence health^13^.

To date, much of the small empirical literature on educational quality and health has focused on cognitive health outcomes rather than physical health, despite the well-established association between education and physical health^14,15^. For example, improvements in educational quality at the state-level (e.g., student–teacher ratio) have been associated with reduced risk for dementia^16^, and educational quality assessed using an individually assessed word-reading subtest has been associated with memory decline even after controlling for educational attainment^15^. Current research also tends to focus on differences in educational quality between groups as broad context or policy differences (e.g., funding, length of school days, racial segregation) or examines the implementation of high-quality early childhood education such as Head Start. While these studies inform whether educational quality may be associated with different outcomes across groups of learners, they cannot speak to individual level outcomes or the psychobiological processes that may help explain how educational quality can protect against poor health, perhaps even over and above educational attainment. An individual and objective measure of educational quality might fill such gaps.

### Theoretical mechanisms connecting educational quality to cardiovascular health

Education is thought to influence cardiovascular health through multiple pathways including perceived control, executive functioning, and health literacy^7,17^. Perceived control, the belief that one’s actions can directly influence life outcomes, has been consistently linked to better health, physical functioning, and lower mortality risk^18–20^. Further, perceived control appears to mediate associations between education and poor health, including outcomes of mortality, incident disease, and biological risk factors^21–23^. Education is hypothesized to increase perceived control through the development of analytic and communication skills^24,25^. Self-reported perceptions of control also are more predictive of outcomes than actual control, perhaps because feeling in control is associated with lower psychological stress^10,26^.

Education may also influence cardiovascular health through executive functioning. Executive functioning is a set of higher order cognitive processes that involve reasoning, problem solving, and the planning, organization, and execution of behavior^27–29^. Executive functioning is associated with educational quality^30,31^, and educational quality has been shown to be a better predictor of decline in executive function than educational attainment in an ethnically diverse cohort^32^ (p.215). Deficits in executive functioning have also been implicated in the onset and progression of multiple diseases, including cardiovascular and metabolic diseases, and have been specifically implicated in weight management (for a review see^33^). For example, inhibition, a core component of executive function, is germane to weight management because it is associated with lower risk of overeating^33^.

Lastly, educational quality may also influence cardiometabolic health, in part, through health literacy. Health literacy is the ability to obtain and understand basic health information and services^1^. General literacy and analytic skills are fundamental to the development of health literacy. Greater health literacy is associated with more education and higher educational quality^34,35^ and lower morbidity including from cardiometabolic diseases^36,37^. Poorer health literacy is thought to contribute to poorer health, in part, because it is associated with poorer comprehension of health care services and outcomes as well as poorer health behaviors^38^. Additionally, educational quality appears to be more closely related to objective measures of health literacy than educational attainment^14,15^.

### The current study

The current study tested whether educational quality was associated with BMI and two measures of central adiposity (visceral fat, waist circumference), all of which are risk factors for poor cardiometabolic health^39,40^. We examined whether effects remained after adjustment for demographic factors as well as educational attainment, and we tested whether perceived control, executive function, or health literacy mediated the association between educational quality and our health outcomes.

To validate our findings, two independent samples were used. An exploratory analysis used data from the Alabama Brain Study on Risk for Dementia (ABSoRD)^41^, which had more relevant measures including visceral fat, waist circumference, perceived control, and health literacy. The confirmation analysis used data from the Human Connectome Project-Aging (HCP-A)^42^ and had many overlapping measures (e.g., BMI, education quality, and executive function).

## Methods

### Design and participants

All participants provided written, informed consent and the research was performed as approved by the Institutional Review Board at the University of Alabama. Additionally, all research was performed in accordance with the Declaration of Helsinki. Both data sets were studies that also collected neuroimaging data and excluded middle-aged and older adults with MRI contraindicators, who self-reported any neuropsychological disorders or scored low on dementia screeners, a history of stroke, and traumatic brain injury (for details see ref. 41 and 42). ABSoRD data included 98 participants (62% females; 62% non-Hispanic White) with a mean age of 51.12 years (SD = 17.46) collected from Birmingham, AL. Educational attainment varied considerably in this sample (see Supplemental Table 1), with 34.4% of the sample reporting a high school degree or less, 16.7% an associate’s degree, 33.3% a bachelor’s degree, and 15.6% a graduate degree. The HCP-A data included 586 participants (59% females; 65% non-Hispanic White) with a mean age of 58.10 (SD = 13.85) collected from four sites: St. Louis, MI, Minneapolis, MN, Boston, MA, and Los Angeles, CA. Educational attainment also varied considerably in this sample, ranging from 7 to 21 years of education, though almost three-fourths of participants (72.35%) reported more than 16 years of education (see Supplemental Table 2).

### Measures

#### Educational quality

Consistent with past research^8,43–47^, educational quality was measured by oral word reading performance using the WRAT-4 in ABSoRD^48^ or the Oral Reading Recognition Test from the NIH Toolbox^49^. Up to 55 English words of various difficulty were read aloud by participants. Word reading has been validated as a measure of education quality by showing high correlations with a self-reported Quality of Education Scale^50^ and with objective measures of student funding, student–teacher ratio, and days of school^43^.

#### Educational attainment

In the exploratory analyses in the smaller sample, educational attainment was operationalized using six levels: primary school, high school, associate's degree, bachelor's degree, master's degree/MBA/JD, doctoral degree. In the larger confirmation sample, years of education was used, which also extends the generalizability of educational attainment.

#### BMI, waist circumference, and visceral fat

Height was self-reported by participants. Both weight and visceral fat (fat surrounding internal organs) were assessed using an OMRON body composition BF511 scale (Omron, Japan), which uses bioelectrical impedance to measure visceral fat. BMI was calculated as weight (kg) / height (m)^2^. Waist circumference was assessed by trained study staff who measured circumference at the level of the navel.

#### Perceived control

Perceived control was only measured in the ABSoRD study, and was measured using the internal control subscale of the Locus of Control Scale (LOC)^51^. This subscale consists of eight items, each on a 6-point likert scale and has demonstrated reliability and validity ^52^. Items included “Whether or not I get to be a leader depends mostly on my ability.”

#### Executive function

Executive functioning was measured using the same two assessments in both the ABSoRD and HCP-A samples: the Trail Making Test—part B (TMT-B) and the Flanker from the Attention Network Test (ANT). The TMT-B is designed to assess frontal lobe functioning, and is a frequently used neuropsychological test of executive function that records participants’ speed alternating between several numbers and letters. The TMT-B measures one major component of executive functions—switching. The TMT-B has demonstrated adequate reliability and validity^53^. The Flanker measures another major component of executive functioning—response inhibition^54^. The task intersperses predictive and misleading visual cues. In this task, participants are asked to press a key on the keyboard to indicate whether a center arrow is pointing to the left or right as fast as they can. On some trials, several arrows to the left and right of the central arrow also are presented that are either in the same direction as the central arrow (congruent trials) or in the opposite direction (incongruent trials). Participants must inhibit the tendency to respond in line with the flanking arrows in the incongruent trials, usually resulting in a slowing down of response times. An executive attention score is created by subtracting reaction times in the congruent condition from reaction times in the incongruent task such that higher scores represent a greater difficulty inhibiting the flanking arrows (i.e., poorer performance). This measure of the Flanker has been shown to have a split-half reliability of 0.70^55^.

#### Health literacy

Health literacy was only measured in the ABSoRD study, and measured with the Health and Financial Literacy Assessment^56^. The nine questions from the health literacy section were used, testing participants’ knowledge about Medicare, doctor’s prescription instruction, drug risk information, and leading cause of death in older adults. The nine multiple-choice health literacy questions have shown adequate internal reliability^56^ and convergent validity^57^. Items included “True or false? Medicare routinely covers costs associated with extended long-term care, such as nursing home care lasting more than 1 month.”

#### Fluid intelligence

In ABSoRD, fluid intelligence was assessed using Raven’s Progressive Matrices (RPM)^58^, and in HCP-A a fluid composite score was used. In ABSoRD, using the RPM, participants viewed a series of patterns with a portion missing and had to choose the pattern that correctly filled the missing portion from eight potential options. Four sets were given with increased difficulty with each set. Only half of each set was used to allow for potential re-use for longitudinal testing, and accuracy was used as the primary outcome variable. In addition, the first one in the set served as practice and was not counted in the final score, leaving a possible total of 23 items. Reliability and validity are good for the RPM. Internal consistency exceeds 0.90^59,60^, and test–retest reliability up to a year is adequate, exceeding 0.85^59^. Correlations with other intelligence tests range from 0.54 to 0.86^59,60^. In HCP-A, a fluid composite score was a precomputed measure that averaged normalized scores from five tasks in the NIH Toolbox: Flanker, Dimensional Change Card Sort, Picture Sequence Memory, List Sorting, and Pattern Comparison^61^. This composite measure has good internal consistency at 0.83, good test–retest reliability at 0.86, and good convergent validity with standard measures (r = 0.78)^62^.

#### Additional covariates

The following demographic variables were included in the models as control variables: age, sex, and ethnoracial category. For the ABSoRD data, ethnoracial category (i.e., race) was coded as White (2), Black/African American (1), and all other identities (0). For the HCP-A data, ethnoracial category was divided into non-Hispanic White, non-Hispanic Asian, non-Hispanic Black, non-Hispanic Other, and Hispanic groups. Age was a continuous variable calculated as the number of years from birth to the date of cognitive testing. Sex was coded as a binary categorical variable; male (0) and female (1).

### Overview of statistical analyses

Bivariate correlations were examined among primary study variables and provide an initial estimate of unadjusted effect size between variables. We also examine partial correlations adjusted for age, sex, and ethnoracial category. Multivariate regression analyses were performed to examine unique associations between objectively assessed educational quality and outcomes. Regressions are presented in a hierarchical format, with the first model controlling for demographic covariates (age, sex, ethnoracial category), the second model adding educational quality, and the third model examining whether any effect of educational quality remains after controlling for educational attainment. Sensitivity analyses further controlled for fluid intelligence as an important potential confound in both samples. The viability of mediators was also tested using regression; first verifying that the predictor variable is significantly associated with the mediator and that the mediator is significantly associated with the outcome variable as theoretically hypothesized. The potential mediators of perceived control and health literacy were only available in the ABSoRD data, and two identical measures of executive function were available in both data sets.

**Table 1:** Correlations among primary study variables and descriptive statistics for each variable.

	Income	Education attainment	Education quality	Internal LOC	Trails B^a^	Flanker	Health literacy	BMI	Visceral Fat	WC
Income	–	**.13**	.29*	**.06**	**−.15**	**−.08**	**.17**	**.04**	**.10**	**.11**
Education attainment	.23^ƚ^	–	.50*	**.10**	−.21*	**−.18**	**.18**	**−.17**	**−.14**	**−.17**
Education quality	.18	.49*	–	**.14**	**−.18**	−.26*	.36*	−.32*	−.24*	−.24*
Internal LOC	.08	.02	.07	–	−.27*	−.22*	.23*	−.27*	−.27*	−.26*
Trails B	−.23	−.17	−.11	−.19	–	.37*	−.**16**	**.05**	**.20**^ƚ^	**.21**^ƚ^
ANT Flanker	−.02	−.22	−.23	−.19	.32*	–	−.**13**	.27*	.30*	.27*
Health literacy	−.09	.19	.28*	.20	−.14	−.15	–	−.**07**	−.**10**	−.**11**
BMI	.07	−.10	−.27*	−.30*	−.05	.11	−.04	–	.82*	.78*
Visceral Fat	.11	−.05	−.22	−.23	−.02	.05	−.02	.88*		.77*
WC	.15	−.10	−.23	−.29*	.01	.05	−.16	.75*	.71*	
*n*	87	96	98	91	86	95	94	98	98	98
*M*	4.12	3.29	104.60	4.51	73.37	158.49	6.74	27.60	9.35	100.04
*SD*	2.37	1.16	17.10	.83	29.05	69.40	1.92	5.99	5.01	18.31
Range	1–8	1–6	64–141	1.00–6.00	27.53–168.68	−11.00–401.00	0–9	19.10–50.30	2–25	33–139

Bolded values above the diagonal are bivariate correlations and those below the diagonal are partial correlations adjusted for age, sex and ethnoracial category. WC = waist circumference.

^ƚ^*p* < .06, *p < .05, a = one outlier windsorized for analyses.

## Results

### Descriptive analyses and correlations

Descriptive statistics as well as bivariate correlations and partial correlations adjusting for demographic variables (age, sex, ethnoracial category) are presented in Tables 1 and 2. In the smaller ABSoRD dataset (Table 1), educational attainment and educational quality were correlated near 0.50 whether unadjusted or adjusted for demographic variables. BMI, visceral fat, and waist circumference were all highly correlated, all *r* > 0.71, all *p* < 0.001 whether unadjusted or adjusted for demographic variables. All three outcome variables were similarly and significantly correlated with educational quality in bivariate analyses (all *r* between −0.32 and −0.24, all *p* < 0.001). Educational quality was not associated with perceived control or executive function as indexed by Trails B, but was significantly associated with executive function as indexed by the Flanker and health literacy in bivariate correlations. In bivariate analyses, perceived control and executive function as indexed by the Flanker was significantly associated with all three outcome variables, executive function as indexed by Trails B was marginally associated with visceral fat and waist circumference (both *p* < 0.06), and health literacy was not associated with any outcome variable. In the HCP-A data (Table 2), correlations were overall weaker. BMI was only correlated with years of education and educational quality before covariates were added into the model. BMI was not associated with Flanker or Trails B performance.

**Table 2:** Correlations among primary study variables and descriptive statistics for each variable in the HCP-A data set.

	Education attainment	Education quality	Fluid Cognition	Flanker	Trails B^a^	BMI
Education attainment	–	.39*	.23*	.23*	−.23*	−.16*
Education quality	.36*	–	.34*	.27*	−.38*	−.15*
Fluid Cognition	.25*	.29*	–	0.73*	−.60*	−.**04**
Flanker	.21*	.21*	.64*	–	−.45*	−.**02**
Trails B^a^	−.21*	−.31*	−.32*	−.32	–	**.08**
BMI	−.10	−.12	−.03	−.03	.08	–
*n*	586	586	586	586	586	586
*M*	17.50	110.32	99.88	97.43	72.29	27.15
*SD*	2.19	14.68	12.03	7.99	38.21	4.90
range	7–21	66–146	61–137	58–117	18.54–317.71	15.66–43.75

Bolded values above the diagonal are bivariate correlations and those below the diagonal are partial correlations adjusted for age, sex and ethnoracial category.

^ƚ^*p* < .06, *p < .05, a = one outlier windsorized for analyses.

### Is educational quality associated with BMI and central adiposity?

Table 3 shows results of regression analyses predicting BMI, visceral fat, and waist circumference. In demographic models predicting BMI, age was the only demographic variable significantly associated with BMI. When educational quality was added in Model 2, it was significantly associated with BMI. The addition of educational quality also doubled the predictive utility of the model as indexed by the change in R^2^ from Model 1 and this was a significant difference; F-change (1, 91) = 8.62, *p* = 0.004. As seen in Table 4, the same results were found for BMI in the HCP-A sample; F-change (1, 216) = 9.64, *p* = 0.002. Further adjusting for educational attainment in Model 3 did not significantly increase R^2 ^and educational quality continued to be similarly and significantly associated with BMI.

**Table 3:** Results of regression models predicting BMI, visceral fat and central adiposity in the ABSoRD sample.

Model & predictor variables	BMI					Visceral fat					Waist circumference				
	beta [95% CI]	*B*	*F/∆F*	*df/∆df*	*R*^*2*^	beta [95% CI]	*B*	*F/∆F*	*df/∆df*	*R*^*2*^	beta [95% CI]	*B*	*F/∆F*	*df/∆df*	*R*^*2*^
**Model 1: Demographics**			2.34*	3	.07			13.70*	3	.31			6.26*	3	.17
Age	.09 [.02, .16]	.26*				.14 [.08, .19]	.47*				.41 [.21, .61]	.39*			
Sex	.41 [−2.09, 2.90]	.03				−2.98 [−4.78, −1.18]	−.29*				−5.30 [−12.54, 1.93]	−.14			
Race	−1.14 [−2.89, .60]	−.14				−.97 [−2.23, .29]	−.14				−1.71 [−6.76, 3.35]	−.07			
**Model 2: + Edu Quality**			9.71*	1	.16			7.66*	1	.36			7.86*	1	.24
Age	.08 [.01, .15]	.23*				.13 [.08, .18]	.45*				.39 [.19, .59]	.37*			
Sex	−.08 [−2.48, 2.33]	−.01				−3.30 [−5.05, −1.55]	−.32*				−6.58 [−13.62, .46]	−.17			
Race	−.11 [−1.90, 1.68]	−.01				−.30 [−1.61, 1.00]	−.04				1.01 [−4.24, 6.26]	.04			
Educational Quality	−.11 [−.19, −.04]	−.32*				−.07 [−.13, −.02]	−.25*				−.30 [−.51, −.09]	−.28*			
**Model 3: + Edu Attainment**			.05	1	.16			.00	1	.36			.06	1	.24
Age	.08 [.01, .15]	.24*				.13 [.08, .18]	.45*				.39 [.19, .58]	.37*			
Sex	−.05 [−2.48, 2.38]	.00				−3.30 [−5.07, −1.53]	−.32*				−6.67 [−13.58, .45]	−.18			
Race	−.11 [−1.91, 1.70]	−.01				−.31 [−1.62, 1.01]	−.04				.99 [−4.29, 6.27]	.04			
Educational Quality	−.12 [−.20, −.04]	−.34*				−.07 [−.13, −.01]	−.25*				−.29 [−.53, −.04]	−.26*			
Educational Attainment	.13 [−1.03, 1.30]	.03				−.01 [−.86, .84]	.00				−.42 [−3.82, 2.99]	−.03			

*p < .05.

**Table 4:** Results of regression models predicting BMI in HCP−A data set.

Model & predictor variables	Beta [95% CI]	*B*	*SE*	*F/*Δ*F*	*df/*Δ*df*	*R*^*2*^ for model
**Model 1: Demographics**				6.48***	6	.06
Age	−.03* [−.06, −.003]	−.10	.02			
Sex	.50 [−.29, 1.29]	.10	.40			
Non-Hispanic Asian	−4.36** [−7.40, −1.32]	−.24	1.55			
Non-Hispanic Black	1.97** [.80, 3.14]	.14	.60			
Non-Hispanic Other	1.78 [−.98, 4.53]	.11	1.40			
Hispanic	0.67 [−.63, 1.98]	.04	.67			
**Model 2: + Edu Quality**				9.64**	1	.08
Age	−.04** [−.07, −.01]	−.11	.02			
Sex	.54 [−.24, 1.32]	.11	.40			
Non-Hispanic Asian	−4.35** [−7.37, −1.33]	−.24	1.54			
Non-Hispanic Black	1.35* [.12, 2.57]	.10	.63			
Non-Hispanic Other	1.53 [−1.21, 4.28]	.09	1.40			
Hispanic	.33 [−.99, 1.64]	.02	.67			
Educational Quality	−.04** [−.07, −.02]	−.13	.01			
**Model 3: + Edu Quantity**				2.59	1	.08
Age	−.04* [−.07, −.01]	−.11	.02			
Sex	.57 [−.21, 1.35]	.12	.40			
Non-Hispanic Asian	−4.05** [−7.09, −1.01]	−.22	1.55			
Non-Hispanic Black	1.29* [.06, 2.51]	.09	.63			
Non-Hispanic Other	1.45 [−1.29, 4.19]	.09	1.40			
Hispanic	.23 [−1.09, 1.55]	.01	.67			
Educational Quality	−.04* [−.07, −.01]	−.07	.02			
Education Attainment	−.16 [−.36, .04]	−.11	.10			

*p < .05, **p < .01.

Models predicting visceral fat showed a very similar pattern of results. In Model 1, sex was a significant predictor of visceral fat in addition to age. In Model 2, educational quality was significantly associated with visceral fat, and adding educational quality to the model significantly improved the predictive utility of the model; *F*-change (1, 91) = 7.00, *p* = 0.010. In Model 3, educational quality remained similarly and significantly associated with visceral fat even after adjustment for educational attainment.

Models predicting waist circumference also showed a similar pattern of results. In Model 1, age was the only demographic factor significantly associated with waist circumference. In Model 2, educational quality was significantly associated with waist circumference, and adding educational quality to the model significantly improved the predictive utility of the model; *F*-change (1, 91) = 6.42, p = 0.013. In Model 3, educational quality remained similarly and significantly associated with visceral fat after adjustment for educational attainment.

### Do differences in subjective perceptions of control, executive function, or health literacy help explain the association between educational quality and outcomes?

Perceived control was only available in the smaller ABSoRD dataset. In multivariate regression analyses, perceived control was significantly associated with BMI (*b* = −1.90, *SE* = 0.78, *p* = 0.018), visceral fat (*b* = −1.27, *SE* = 0.58, *p* = 0.032), and waist circumference (*b* = −5.28, *SE* = 2.26, *p* = 0.022) after adjusting for age, ethnoracial category, and sex. However, educational quality was not significantly associated with perceived control after adjusting for age, ethnoracial category, and sex. Given the lack of association between the predictor and the proposed mediator, we did not perform further tests of mediation. Interestingly, when educational quality and perceived control are entered into models simultaneously both are independent predictors of all three outcome variables.

The same two measures of executive function, Trails B and the Flanker task, were available in both samples. Neither measure of executive function was significantly associated with any of the three outcome variables after adjusting for age, ethnoracial category, and sex in either sample. Given the lack of association between this proposed mediator and the outcomes, we did not perform further tests of mediation.

Health literacy was only available in the smaller ABSoRD dataset. Health literacy also was not significantly associated with any of the three outcome variables after adjusting for age, ethnoracial category, and sex. Given the lack of association between this proposed mediator and the outcomes, we did not perform further tests of mediation.

### Sensitivity analysis

Like other cognitive assessments, the measure of educational quality used here may also be influenced by fluid intelligence (e.g., general aptitude). Given this, we examined whether educational quality was significantly associated with cardiometabolic health after adjustment for fluid intelligence: RPM from the smaller exploratory sample and a fluid composite score from HCP-A. Results are shown in Table 5. Educational quality remained a statistically significant predictor of BMI, visceral fat, and waist circumference. Fluid intelligence was not a significant predictor of any of the three outcomes. Effect sizes (standardized betas) were statistically indistinguishable from models without adjustment for fluid intelligence (Tables 3 and 4, Model 2).

**Table 5:** Regression models of educational quality predicting BMI, visceral fat, and central adiposity controlling for fluid intelligence.

Predictor variables	BMI		Visceral Fat		Waist Circumference		BMI (HCP-A)	
	beta [95% CI]	*B*	beta [95% CI]	*B*	beta [95% CI]	*B*	beta [95% CI]	*B*
Age	.09 [.02, .16]	.25*	.13 [.073, .18]	.43*	.40 [.20, .60]	.38*	−.05 [−.09, −.01]	−.14**
Sex	.16 [−2.27, 2.59]	.01	−3.04 [−4.84, −1.23]	−.30*	−5.60 [−12.73, 1.53]	−.15	.50 [−.28, 1.29]	.10
Race^a^	−.28 [−3.01, 2.46]	−.02	−.11 [−2.15, 1.93]	−.01	.14 [−7.90, 8.19]	.01		
Non-Hispanic Asian							−4.39 [−7.41, −1.37]	−.24**
Non-Hispanic Black							1.17 [−.12, 2.45]	.08
Non-Hispanic Other							1.56 [−1.19, 4.30]	.09
Hispanic							.24 [−1.09, 1.57]	.02
Fluid Intelligence	.13 [−.21, .46]	.08	−.01 [−.26, .25]	−.01	.02 [−.99, 1.02]	.01	−.02 [−.06, .02]	−.05
Educational Quality	−.12 [−.20, −.04]	−.34*	−.07 [−.13, −.02]	−.25*	−.27 [−.50, −.04]	−.25*	−.04 [−.07, −.01]	−.12**

Fluid intelligence was assessed using the Raven's Progressive Matrices task. *p < .05; ^a^Race was coded as Non-Hispanic White vs. other

## Discussion

Most prior studies examining educational health disparities have assessed educational attainment, either by years of education or highest degree attained. Educational quality may be more closely related to physical health because it directly assesses the cognitive and skill-based benefits of education (e.g., knowledge, skills, reasoning, effectiveness) that are thought to confer benefits to health^8^. This study examined whether an objective individual-level assessment of educational quality, which has been widely used in studies of education quality and cognitive health, was associated with cardiometabolic health. Analyses further examined whether perceived control, performance-based assessments of executive function, or health literacy may explain the association between educational quality and cardiometabolic health. We originally performed analyses in a smaller exploratory dataset (ABSoRD) and then a second, larger sample was used to replicate the findings when conceptually similar variables were available. The two samples complemented each other in many respects. For example, ABSoRD had greater variability in educational attainment than the larger HCP-A dataset, and constructs were often operationalized using different measures across studies.

**Figure 1:**
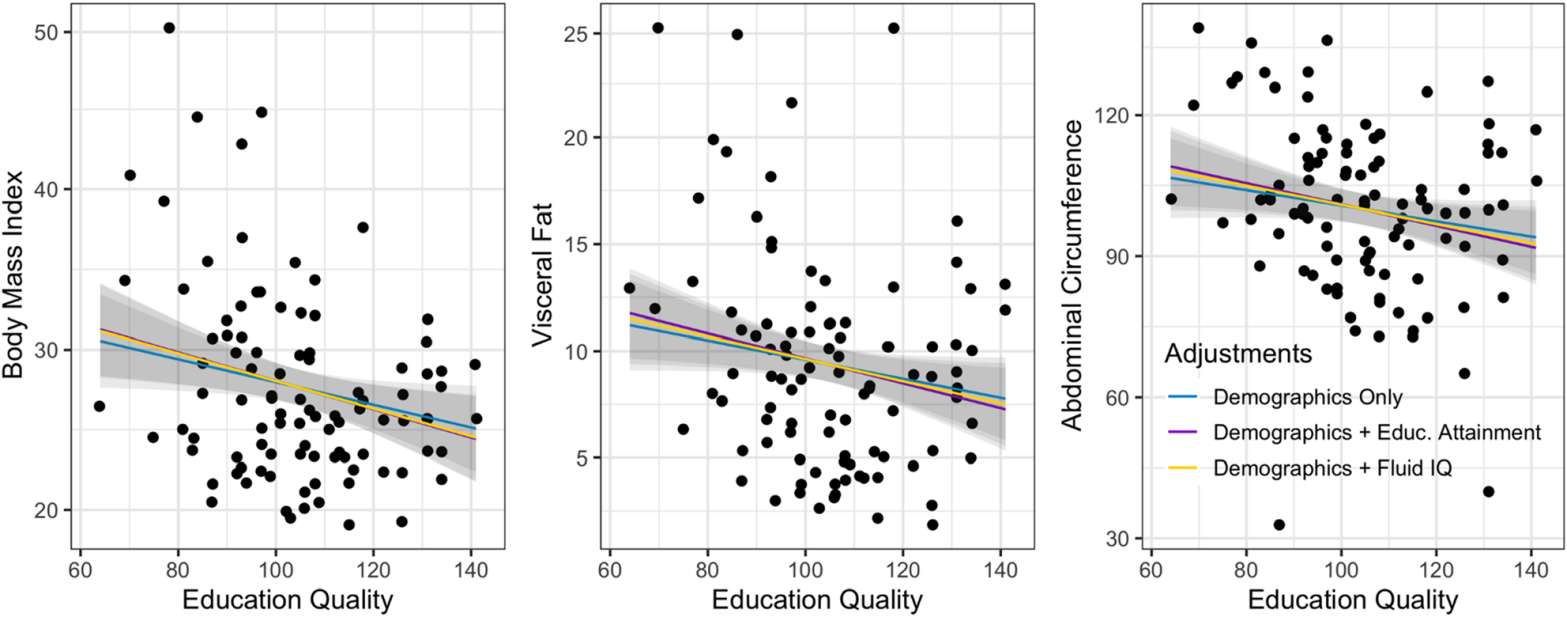
Associations between educational quality and BMI, visceral fat, and waist circumference. The blue line is the association between educational quality and the outcome adjusting for demographics (age, sex, ethnoracial category). The purple line further adjusts for educational attainment as measured by educational attainment. The yellow line further adjusts for fluid IQ. The strength of the association between educational quality and outcomes is virtually unchanged after adjustment for demographics, educational attainment, or fluid IQ.

**Figure 2:**
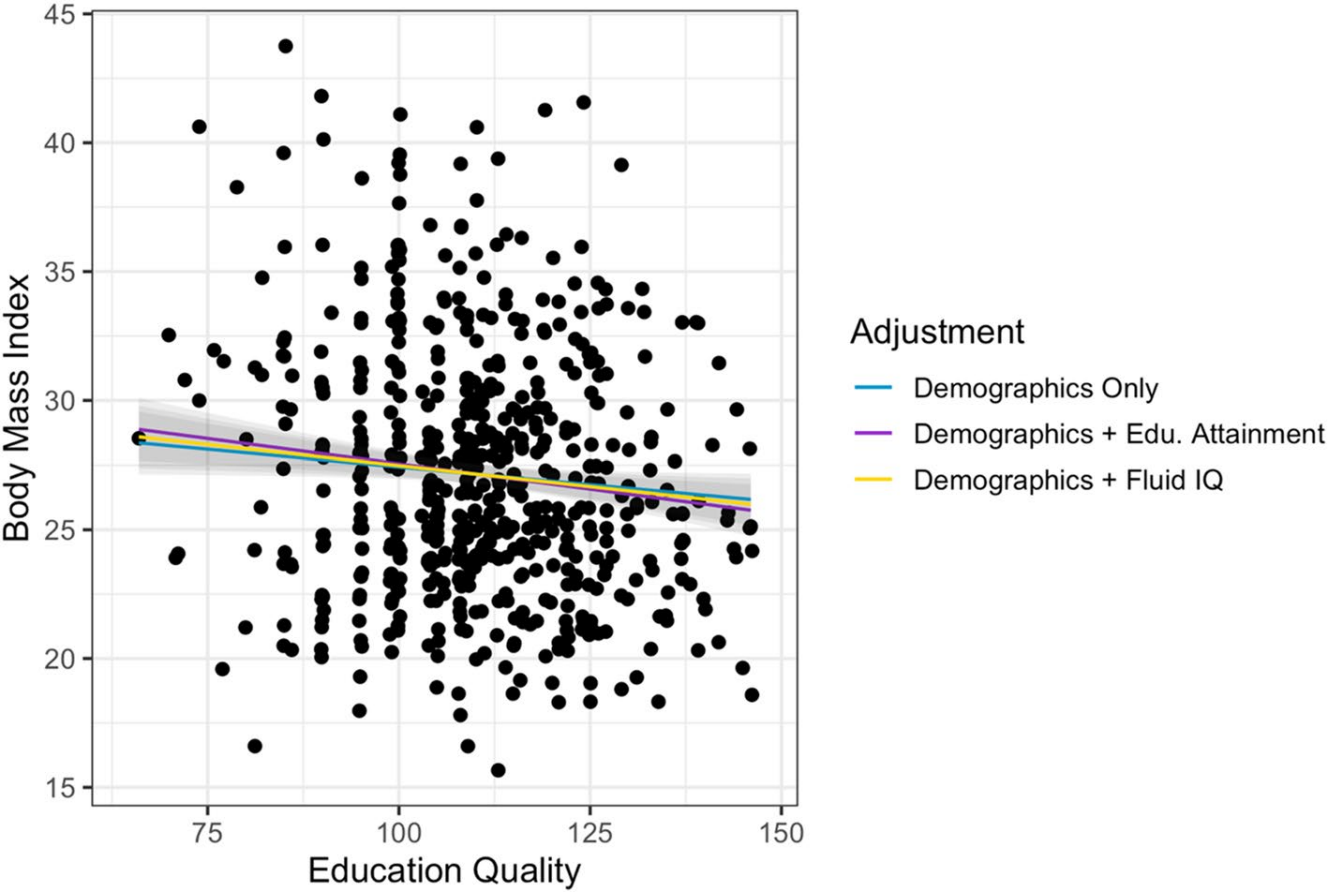
Associations between educational quality and BMI in the HCP-A data set. The blue line is the association between educational quality and the outcome adjusting for demographics (age, sex, ethnoracial category). The purple line further adjusts for educational attainment as measured by educational attainment. The yellow line further adjusts for fluid IQ. The strength of the association between educational quality and outcomes is virtually unchanged after adjustment for demographics, educational attainment, or fluid IQ.

Initial correlations suggested that educational quality was a closer correlate of cardiometabolic health. In the ABSoRD dataset, educational quality (v. educational attainment) was equally or more strongly associated with all outcomes and mediators with the exception of Trails B. In the HCP-A dataset, which also collected Trails B, educational quality was equally or more strongly associated with all outcomes and mediators. Despite the moderate correlation between educational attainment and educational quality (~ *r* = 0.50 and ~ *r* = 0.40 for the exploratory and confirmatory samples, respectively), the relationship between educational quality and cardiometabolic health was independent of educational attainment in both samples. The association between educational quality and cardiometabolic health was also unchanged after adjusting for fluid intelligence in both samples (for a visual comparison of the association between educational quality and outcomes using progressive statistical adjustments see Figs. 1 and 2). Thus, associations between cardiometabolic health and educational quality cannot be attributed to underlying differences in general aptitude (e.g., educational quality is not just a proxy for aptitude). These findings contribute additional supporting evidence to the small literature showing that educational quality may be more closely associated with physical health than educational attainment or quantity (e.g., years in school, highest degree attained).

While associations between educational quality and cardiometabolic health outcomes were highly consistent, we did not find support for the mediating role of perceived control (smaller exploratory sample only), two different measure of executive function (both samples), or health literacy (smaller exploratory sample only). Perceived control is frequently assessed in research examining the association between socioeconomic status and physical health^23,63,64^. Consistent with this literature, greater perceived control was associated with better cardiometabolic health, even though it did not mediate associations between education and cardiometabolic health. Although education may sometimes increase perceived control, the present study suggests that other factors, at least in the current sample, might have influenced one’s perceived control. For example, having a larger family structure and the social support that family can bring has been shown to be related to perceived control^65^. Lifetime experiences with healthcare systems, stressors, and social pressures also can impact one’s level of perceived control, which could also affect coping behaviors related to both the physiology underlying eating behaviors (e.g., satiety responses) as well as psychological components of eating to regain feelings of control^66^. Many of these factors are independent of educational attainment or quality in their impact on cardiometabolic health.

Executive function was assessed using two tasks: one assessing mental switching (Trails Making B, both samples) and another representing response inhibition (Flanker, both samples). Although we found some marginal associations between executive function, education, and cardiometabolic health before adjustment for demographic variables, no significant associations remained after demographic adjustments in either the exploratory or confirmatory samples. Thus, findings did not warrant mediation analyses. In the larger confirmatory sample, the two executive function measures were unrelated to BMI regardless of whether demographic variables were adjusted.

Health literacy was assessed by objective assessment of participants’ knowledge regarding health and healthcare. The measurement and importance of health literacy for cardiometabolic health and health disparities has recently garnered significant attention, in part, because health literacy is low in the United States and is associated with cardiometabolic health^67,68^. Health literacy was significantly associated with educational quality but not educational attainment in this sample but was not associated with cardiometabolic health (Table 1). The measure of health literacy used here was originally developed in a sample of older adults and contained several questions targeting this age group (e.g., What is Medicare?). Future research using health literacy assessments specific to lifelong health matters or cardiometabolic health may be more sensitive.

### Limitations

This study has many methodological strengths that make it an important contribution to the literature such as objective assessment of both independent and dependent variables and examination of two distinct diverse community samples often with differing operationalization of constructs. Further, in-person objective assessments such as those used here can reduce measurement noise compared to larger national data sets in which constructs are assessed via telephone interviews and less reliable measures are used^26,69^. However, interpretation of results should also consider study limitations. Neither study samples were representative of the population, both were collected in urban locations and both samples consisted primarily of middle-aged and older adults. Although participants did vary considerably in educational attainment, most participants in the larger HCP-A sample reported high levels of education (> 16 years). Thus, results could be interpreted to mean that educational quality is able to differentiate among individuals with similar levels of education. We note, however, that we saw similar incremental utility for educational quality in the ABSoRD sample who did not tend to be highly educated and had more variability in educational attainment. Additionally, although the exploratory sample was not large, we supplemented many of the analyses with a much larger and independent sample that replicated the main findings where possible, despite also being collected from different parts of the country. The potential mediators of health literacy and perceived control were only available in the smaller ABSoRD study, and findings need to be replicated in larger datasets to assess whether mediational analyses may be warranted. Additionally, while we found non-existent to weak correlations with executive functions in both samples. Both executive function tasks examined here used response times to index executive function. Executive functions are very heterogeneous and this does not preclude that other types of executive functions might serve as mediators between education and cardiometabolic health including emotional regulation, working memory updating, and goal-maintenance, to name a few^54,70^. Measures of executive function that capture lasting control of one’s behaviors to both promote health might serve as a more sensitive mediator of cardiometabolic health than the reaction time measures used here.

## Conclusions

Education is one indicator of socioeconomic status that has been consistently associated with multiple health outcomes, including cardiovascular and metabolic health. To date, researchers have primarily assessed education by asking people how many years they attended formal education or their highest attained degree. The present findings suggest that such measures of educational attainment underestimate the association between education and cardiometabolic health. Educational quality may be more closely associated with cardiometabolic health, similar to findings for cognitive health. We do not find support for the hypothesis that executive function, perceived control, or health literacy partially explain the association between educational quality and cardiometabolic health, and executive function was assessed in both samples. These findings have implications for interventions and government policies that aim to improve cardiometabolic health. To the extent that educational programs may have been ignored due to an apparent small relationship between education and cardiometabolic health, such actions may have been premature given that effect sizes for education quality can be appreciably larger than for education attainment even after controlling for demographic factors (Table 3). Although perhaps obvious, programs ensuring high quality educational outcomes is critical, as large variability in educational quality can exist within similar years of schooling or obtaining a similar degree.

## Supplementary Information

Supplementary Information.

## Data Availability

The ABSoRD dataset used and/or analysed during the current study available from the corresponding author on reasonable request. The HCP-A data generated and/or analysed during the current study are available in the Lifespan Human Connectome Project Aging Study repository, https://www.humanconnectome.org/study/hcp-lifespan-aging. The NDA Study record can be found at 10.15154/1528124.
