# Supplementary material for: Educational quality may be a closer correlate of cardiometabolic health than educational attainment
Source: Sci Rep. 2022 Oct 27;12:18105. doi: 10.1038/s41598-022-22666-3 (PMC9613691; doi:10.1038/s41598-022-22666-3)
Supplement: Supplementary file 1 — Supplementary Information. [file 41598_2022_22666_MOESM1_ESM.docx]

Supplemental Table 1. Frequencies and Percentages of the Categories for each Categorical Variables in the ABSoRD data set

| Variables and Categories | Number of Participants | Percentage |
| --- | --- | --- |
| Race |  |  |
| African-American | 25 | 25.5 |
| American Indian/Alaskan Native | 1 | 1.0 |
| Hispanic | 2 | 2.0 |
| Bi-racial or Multi-racial | 6 | 6.1 |
| Iranian | 1 | 1.0 |
| South Asian | 2 | 2.0 |
| White | 61 | 62.2 |
| Sex |  |  |
| Male | 37 | 37.8 |
| Female | 61 | 62.2 |
| Years of Education |  |  |
| Primary School | 2 | 2.1 |
| High School | 31 | 32.3 |
| Associate’s | 16 | 16.7 |
| Bachelor’s | 32 | 33.3 |
| Master’s/ MBA/ JD | 14 | 14.6 |
| PhD/ MD | 1 | 1.0 |

Supplemental Table 2. Frequencies and Percentages of the Categories for each Categorical Variables in the HCP-A

| Variables and Categories | Number of Participants | Percentage |
| --- | --- | --- |
| Race |  |  |
| African-American | 83 | 14.16 |
| American Indian/Alaskan Native | 2 | 0.34 |
| Hispanic | 65 | 11.09 |
| Bi-racial or Multi-racial | 10 | 1.71 |
| Asian | 46 | 7.85 |
| White | 379 | 64.68 |
| Sex |  |  |
| Male | 250 | 42.66 |
| Female | 336 | 57.34 |
| Years of Education |  |  |
| 7 | 1 | 0.17 |
| 8 | 1 | 0.17 |
| 9 | 1 | 0.17 |
| 10 | 3 | 0.51 |
| 11 | 2 | 0.34 |
| 12 | 4 | 0.68 |
| 13 | 30 | 5.12 |
| 14 | 3 | 0.51 |
| 15 | 72 | 12.29 |
| 16 | 45 | 7.68 |
| 17 | 29 | 4.95 |
| 18 | 194 | 33.11 |
| 19 | 143 | 24.40 |
| 20 | 20 | 3.41 |
| 21 | 38 | 6.48 |
